# Inequalities in large‐scale breastfeeding programmes in Bangladesh, Burkina Faso and Vietnam

**DOI:** 10.1111/mcn.13687

**Published:** 2024-07-17

**Authors:** Tina G. Sanghvi, Deepali Godha, Edward A. Frongillo

**Affiliations:** ^1^ Alive & Thrive initiative, FHI 360, Family Health International, Washington DC and Durham North Carolina USA; ^2^ Consultant FHI 360, 406 Ghanshyam Castle, Khajrana Square Indore Madhya Pradesh India; ^3^ Department of Health Promotion, Education, and Behaviour University of South Carolina Columbia South Carolina USA

**Keywords:** Bangladesh, breastfeeding, Burkina Faso, counselling, inequalities, mass media, Vietnam

## Abstract

Inequalities in breastfeeding programmes and practices have slowed global progress in providing the life‐saving protection of breastfeeding for millions of infants despite well‐known life‐long impacts. As breastfeeding interventions are scaled up, inequalities in coverage and breastfeeding practices should be tracked, particularly in disadvantaged groups, who are likely to suffer the most serious health and developmental impacts of poor childhood nutrition. The literature provides evidence of inequalities in breastfeeding practices, but research is limited on socioeconomic disparities in the coverage of breastfeeding interventions. This paper (1) compares inequalities in breastfeeding practices in intervention and nonintervention areas and (2) documents inequalities in programme coverage by type of intervention. We disaggregated endline evaluation surveys in Bangladesh, Burkina Faso and Vietnam, where rigorous evaluations had documented significant overall improvements, and analysed whether inequalities in breastfeeding practices and programme coverage differed by treatment areas. We used Erreygers index to quantify inequalities and found that breastfeeding practices were largely pro‐poor; intervention coverage was not consistently pro‐poor. While counselling coverage often favoured women from the poorest quintile, public education/media coverage consistently favoured better‐off women. Inequalities favoured more educated mothers in the coverage of combined interventions. None of the programmes had explicit equality objectives. The results indicate the need for introducing specific actions to reduce inequalities in breastfeeding policies and programmes. This is a priority unfinished agenda for nutrition programming.

## Key messages

- Exclusive breastfeeding and early breastfeeding initiation were found to be higher in intervention areas. Exclusive breastfeeding was pro‐poor in all intervention areas. Early initiation and colostrum feeding were pro‐poor in Bangladesh and Vietnam but not in Burkina Faso.
- Exposure to breastfeeding interventions was higher in intervention areas in all countries. While coverage was slightly pro‐poor for interpersonal communication, public education and mass media favoured the better‐off and more educated mothers, although less so in intervention areas.
- None of the programmes had explicit equality objectives. Development of targeted interventions to reduce socioeconomic inequalities remains an unfinished agenda for breastfeeding programmes.

## Graphical abstract

While breastfeeding practices were pro‐poor at endline, intervention coverage did not favour socioeconomically disadvantaged mothers in intervention areas of three large‐scale programmes implemented in LMICs. This indicates a need for specific actions to reduce inequalities in breastfeeding policies and programmes and remains an unfinished agenda for nutrition programming.

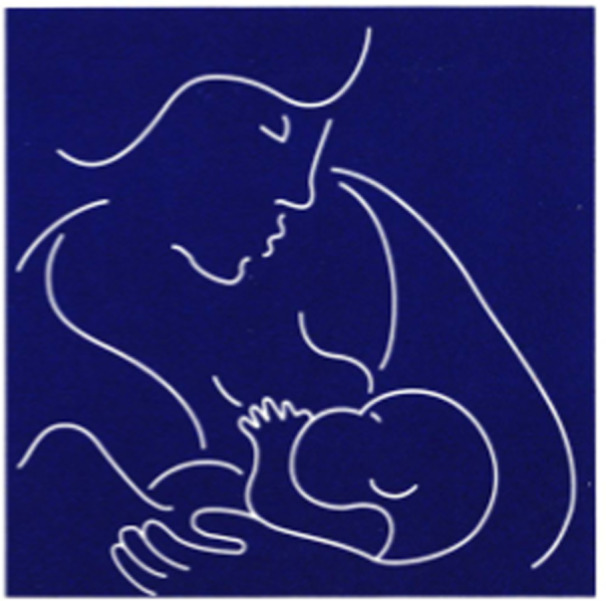

## 1 INTRODUCTION

Efforts to support breastfeeding (BF) practices among mothers of infants can deliver large payoffs in health care savings, improved women's health, health during childhood and adulthood and developmental indicators (Victora et al., 2016; Walters et al., 2019). Yet, almost half the global population of mothers and infants are unable to protect BF (Rollins et al., 2016; Victora et al., 2015, 2016). During 2010–2018, the global weighted prevalence of BF was calculated to be 51.9% for early initiation of BF and 45.7% for exclusive BF under 6 months (Zong et al., 2021). Due to the urgency of accelerating progress in BF, it is a central part of the 2030 Agenda for Sustainable Development (WHO, 2014, 2020). While progress in raising the prevalence of BF is slow, more infants are receiving suboptimal commercial milk formula (Rollins et al., 2023). The impact of replacing BF among disadvantaged mothers and infants is particularly deleterious due to high product costs, tendency to overdilute or switch over to more undesirable products and lack of water and sanitation for safe preparation (Andresen et al., 2007; Caruso et al., 2023; Rothstein et al., 2021). The potential negative impacts of replacing BF on the environment and climate change have been documented (Cerceo et al., 2024). A first step in understanding what needs to be done to protect BF in disadvantaged groups is to document the patterns of inequality in BF trends and coverage of intervention programmes.

Globally recommended evidence‐based BF interventions include counselling and education for mothers, supportive legislation and a mix of channels to reach persons with influence in families, communities and health services (WHO, 2021). Studies estimate that education strategies for BF could increase the prevalence of early initiation of BF (EIBF) by 20% (95% confidence interval [CI]: 12%–28%) and exclusive BF (EBF) by over 100% (88%–117%) at 3 months, and by 53% (47%–58%) at 6 months (Lassi et al., 2020; McFadden et al., 2017). The Lancet reviews on BF found that when accompanied by greater community awareness, supportive social norms and protected by regulations for limiting the marketing of replacement products, less restrictive health services and workplace BF support, these interventions can accelerate and sustain desirable BF patterns (Keats et al., 2021; Rollins et al., 2016). While large‐scale implementation of such programme packages in low‐ and middle‐income countries (LMICs) is not widely documented, a few rigorous evaluations have demonstrated significant impacts on BF prevalence (Cresswell et al., 2019; Flax et al., 2022; Menon et al., 2016). These evaluations provide empirical evidence on the feasibility of achieving BF impacts on a large scale in LMICs but do not document if socioeconomically disadvantaged mothers benefited from BF protection. As similar BF interventions are being adapted and expanded, there is an urgent need to assess inequalities in coverage and uptake of recommended practices.

There is evidence of inequalities in BF practices. BF has been more prevalent among LMICs as compared with high‐income countries and among lower socioeconomic groups within LMICs (Victora et al., 2016). Social norms and cultural beliefs appear to play a role alongside maternal education and access to health services where BF may be supported, for example, during antenatal care (ANC) visits, or pose barriers (e.g., delayed initiation in Caesarean childbirth). In the United Kingdom, mothers of Black, Asian, mixed or other ethnic groups, older mothers and those with more education had better BF practices than others (Simpson et al., 2022). Secondary analyses of demographic and health surveys (DHS) in LMICs found that during 2000–2019, women with no formal education had worsening BF indicators compared to women with primary and secondary or higher education (Neves et al., 2021). In Latin America, overall increases in BF were not distributed equally in Bolivia, Brazil, Colombia and Peru, where the improvements in BF duration were lower in socioeconomically disadvantaged subgroups over a 20‐year period (Lutter et al., 2011). In Pelotas, Brazil, as overall BF indicators improved from 1993 to 2015, the recommended practices were adopted by high‐income women earlier and to a greater extent than by women from the poorest quintile (Santos et al., 2019). In Mexico, the prevalence of exclusive BF and any BF declined between 1999 and 2006 among the women from the poorest quintile, improved among the better off and did not change overall (González de Cossío et al., 2013). A recent scoping review of early initiation of BF in Africa documented associations of higher education and wealth status with better practices (Shimizu et al., 2023). In addition to socioeconomic inequalities, geographic inequalities within countries can also undermine progress and account for slow national improvements in BF prevalence. Geospatial mapping of national surveys for 2000–2018 found that Philippines, Brazil and Nigeria are projected to have large geographic disparities, indicating the need for geographic targeting (Bhattacharjee et al., 2021). Studies on interventions to reduce socioeconomic inequalities in health are limited and programme experiences in supporting BF practices equitably are not widely documented in LMICs (Yuan et al., 2014). In the United States, policy and programme interventions for BF were reportedly unable to reduce ethnic and racial inequalities (Rupnicki, 2020; Segura‐Pérez et al., 2021). One study in Belarus documented inequalities in a BF promotion programme modelled on the WHO/UNICEF Baby‐Friendly Hospital Initiative and found slightly increased levels of inequality in intervention areas (Yang et al., 2014). Our paper fills a gap in the literature by comparing wealth and education inequalities in BF practices in intervention and non‐intervention areas, and documents inequalities in programme coverage by type of intervention in Bangladesh, Burkina Faso and Vietnam.

## 2 METHODS

The programmes examined in this study are part of a larger initiative to improve women, infants and children's nutrition (https://www.aliveandthrive.org/en). Each country pursued a comprehensive strategy and implemented a package of interventions at the individual, home, community and facility levels as indicated by evidence in alignment with global findings (Pérez‐Escamilla et al., 2012; Rollins et al., 2016; Sinha et al., 2017). The programmes reached large‐scale coverage mainly by integrating BF interventions into existing health services to directly support women through face‐to‐face counselling and through public education/media channels; structural interventions were in place in Bangladesh and actively pursued in Burkina Faso and Vietnam (Cresswell et al., 2019; Menon et al., 2016). Health systems strengthening was needed at facility and community levels to fit in with service delivery patterns. Content was shaped by behavioural science principles to address underlying motivations and barriers in the adoption of BF practices such as knowledge and beliefs, self‐efficacy, family support and social norms.

### 2.1 Data

The data were from cross‐sectional household surveys containing publicly available deidentified files prepared by the International Food Policy Research Institute (IFPRI) for Bangladesh and Vietnam and the London School of Hygiene and Tropical Medicine (LSHTM) for Burkina Faso. These data are publicly available from the Alive & Thrive website (IFPRI, LSHTM, 2023).

The study population comprised children under 6 months of age, with a final sample size of 998 mother/baby pairs in Bangladesh, 1162 in Burkina Faso and 1002 in Vietnam. The sample sizes were estimated separately in each country using DHS data to obtain baseline prevalence. Sampling was performed to measure impact on exclusive BF for infants in the age group 0–5.9 months using an RCT evaluation design. The literature on effectiveness of behaviour change interventions was used to obtain effect sizes. Further details on sampling and data collection methods have been described in the impact papers for Bangladesh and Vietnam and Burkina Faso (Cresswell et al., 2019; Menon et al., 2016). Primary data collection was conducted originally for impact assessment of the programme interventions and this study analyses wealth and education inequalities related to BF in intervention and nonintervention areas at endline. Endline surveys were conducted in 2014 in Bangladesh and Vietnam after a 4‐year implementation period, and in 2017 in Burkina Faso after a 2‐year implementation period.

### 2.2 Indicators

Several categories of indicators were used for assessing inequalities including BF practices, maternal perceptions, maternal knowledge and BF intervention coverage. Table 1 lists these indicators and the participant characteristics used for the analysis.

**Table 1:** Indicators for breastfeeding practices, maternal perceptions, maternal knowledge, intervention coverage and participant characteristics.

Indicators	Bangladesh	Burkina Faso	Vietnam
**BF practices** a			
Exclusive BF indicates if the child had only breastmilk in the past 24 h or the day before the survey. Early initiation of BF indicates if BF was initiated within the first hour of birth. Fed colostrum indicates if the child was given colostrum. Prelacteal feeding indicates that the child was given something besides breastmilk in the first 3 days of birth.	Same as noted in col. 1	Same as noted in col. 1	Same as noted in col. 1
**Maternal perceptions**			
Perceived social/community norms is a binary variable that indicates if mothers in the community are perceived to follow the recommended BF practices.	Responses: No, none; yes: few; yes: most; yes: all; and don't know. Of these, if the respondent responded with ‘Yes: most’ or ‘Yes: all’ for the following behaviours, then it was considered yes for correct community norms—mothers in her community breastfeed immediately after delivery, do not give prelacteals (honey, mustard oil) and/or breastfeed the child exclusively for 6 months.	Responses: does not agree and agree. The variable indicates if the respondent does not agree that most people in her village think that a mother should not give the colostrum to her baby; give water to her baby; and/or give *bouillie* to her baby.	Responses: mother agreed or strongly agreed with the statement (using a Likert scale) that most important people think that a mother should BF within the first hour of delivery, whether normal or C‐section, that it should continue for at least 12 months and that she should feed only breastmilk and nothing else for the first 6 months. In addition, a disagreement or strong disagreement to the following also indicated the same: giving water, infant formula or semi‐solid food to the baby before 6 months or using a bottle with a nipple for feeding.
Perceived self‐efficacy is the confidence of mothers in following recommended BF practices	Mothers asked if they felt confident about the following: feel confident that you are/will be able to feed child no pre‐lacteals but only colostrum in the first few days after delivery, feel confident that you are/will be able to only breast feed in the first 6 months, feel confident that you are/will be able to sufficiently produce breast milk to meet the child's need in the first 6 months.	Information not available	Mothers ‘agreed’ or ‘strongly agreed’ with the following statements (using a Likert scale): the ‘first milk’ produced by my body is all my newborn needs in the first 24 h after birth, I can refrain from giving my infant water before s/he reaches 6 months of age, I can convince other caretakers of my infant to not give him/her water to drink before s/he reaches 6 months of age.
**Maternal knowledge**			
The total score is the sum of the following six factors, with 1 point for each correct response	Same as noted in col. 1	Same as noted in col. 1	Same as noted in col. 1
Exclusive BF knowledge indicates the respondent's understanding that it means giving no other food or liquids to a child <6 months of age. Importance of EBF for the child indicates that mother knows specific reasons for BF that can be beneficial to the child. Importance of EBF for the mother indicates that the mother knows specific reasons for BF that can be beneficial to herself. Feed‐timing indicates that mother knows that feeding should be done on demand and the child should be fed day and night or fed frequently. Early Initiation indicates that the mother knows that BF should be initiated within the first hour after delivery. Feeding colostrum indicates that the mother knows that colostrum should be fed to the child and not thrown away	Same as noted in col. 1	Same as noted in col. 1	Same as noted in col. 1
**Intervention coverage**			
Interpersonal communication Number of IPC contacts: Mothers were asked if they were visited or they attended services at a facility or group/outreach session where BF practices were discussed. This category is not limited to on‐to‐one individualised counselling but also includes group‐based contacts with trained health workers or facilitators, for example, in Bangladesh at group education sessions and in Burkina Faso at community mobilisation sessions.	Number of times the mother was visited at home by SS, SK and PK during the 7th or 8th month of pregnancy and in the 6 months before the survey but if and only if advice was given on BF during the contact. If the respondent had attended a health forum where BF was discussed, it was also considered a contact	Number of times the respondent participated in a group discussion where BF was discussed or the number of times a health worker visited the respondent's home to advise on BF.	Number of times the mother received nutrition counselling services during pregnancy, at delivery or at MTBT if infant was <6 months (whether it was individual or in a group). Timing was considered (such as pregnancy, infant <6 months, 6 months before the survey). The number of times mothers were visited by a VHW at home during the 6 months before the survey was also added.
Content of IPC interventions: Messages given during IPC: ‐ Initiate BF within 1 h ‐ BF exclusively for 6 months ‐ Feed colostrum. How to position the baby	Same as noted in col. 1	No information available	Same as noted in col. 1
Public education/media exposure	Mother recalled any TV ads in the 3 months before the survey: BF initiation of newborn baby; exclusive BF; BF impact on brain; and animated film on helping mother BF. The binary indicator was 1 if the mother had watched any of the above and 0 otherwise.	Mother recalled hearing about BF on radio since delivery. The binary indicator was 1 if the mother recalled any and 0 otherwise.	Mother recalled seeing or hearing any of the following in the 1 month before the survey and “No” otherwise: any information on BF on television, heard any information on BF on radio, seen any information on BF on the internet. The binary indicator was 1 if the mother recalled any and 0 otherwise.
Exposure to the number of different interventions indicates exposure to both interventions, exposure to IPC was considered a ‘Yes’ if the respondent had received any advice and exposure to media was considered a ‘Yes’ if the respondent had seen/heard any publicly broadcast BF information	Same as noted in col. 1	Same as noted in col. 1	Same as noted in col. 1
**Participant characteristics**			
Child factors ‐ Child's age group: 0–1 months; 2–3 months; 4–5 months. ‐ Sex: male, female ‐ Health facility delivery: yes/no	Same as noted in col. 1	Same as noted in col. 1	Same as noted in col. 1
Maternal factors ‐ Mother's age group: 16–25 years; 26–35 years; 36–55 years. In Burkina Faso, the first category also includes ages 14 and 15 years. ‐ Mother's education: never attended school/in Class 1, completed Classes 1–5 (primary only in Burkina Faso), completed Classes 6–9 and completed Classes 10–12 or higher (secondary or higher in Burkina Faso). ‐ Mother's occupation: housewife, small business, farm work or other. In the Bangladesh study sample, there were no mothers involved in agriculture, while in the Burkina Faso study sample, there were no housewives. ‐ Mother's dietary diversity (MDD) is a score ranging from 0 to 12 based on the number of food groups (total 12) consumed by the mother the previous day or if the previous day was a ‘special’ day on an earlier day <7 days	Same as noted in col. 1	Same as noted in col. 1	Same as noted in col. 1
Paternal and household factors ‐ Father's occupation: Small business, farm work or other. ‐ Household food insecurity has been calculated as per the HFIAS and has four categories: Food secure; mild; moderate, and severe. ‐ Household wealth score has been computed using principal component analysis of household assets and other aspects. This has further been divided into three categories‐ household wealth tertiles, and five categories—household wealth quintiles. These variables were already available in the Burkina Faso data set	Same as noted in col. 1	Same as noted in col. 1	Same as noted in col. 1

Abbreviations: BF, breastfeeding; MTBT, Vietnamese term for franchises; PK, pushti kormi or nutrition worker; SK, shastha kormi or health worker; SS, shastha sebika or community health worker; VHW, village health worker.

^a^ Globally recommended indicators have been used for calculating BF practices.

### 2.3 Statistical analysis

Inequality was measured across the programme participant's wealth and education spectrums in nonintervention and intervention areas. The study used Erreygers index (EI) for binary indicators and concentration index (CI) for continuous indicators to illustrate the nature and extent of socioeconomic (SES)‐related inequalities. The indices quantify the levels of inequality by assessing the distribution of the outcome of interest across the wealth spectrum (wealth inequality) or education spectrum (education inequality) (Erreygers, 2009). EI ranges between −1 and +1, where 0 indicates no inequality, −1 indicates concentration of the outcome among the worse off (poor/uneducated) and +1 indicates concentration among the better off (higher wealth categories/more highly educated). For the wealth spectrum, household wealth scores were constructed using principal components analysis of household assets, whereas the education spectrum was measured as the completed number of years of mothers' education. The inequality indices were estimated using the Stata ‘conindex’ command, with accounting for geographical clustering (StataCorp, 2017).

### 2.4 Ethical statement

Ethical clearance was obtained by IFPRI and the London School of Hygiene and Tropical Medicine—the institutions that administered the surveys and managed the data. Our analyses used anonymized, publicly available databases.

## 3 RESULTS

### 3.1 Participant characteristics

Table 2 illustrates the diverse contexts in study countries as seen in the variations across descriptive variables for child, parent and household characteristics. Most of the infants were two or more months of age. Bangladesh had the lowest proportion of institutional deliveries. Mothers in Bangladesh were younger than those in Burkina Faso and Vietnam, and more of them were housewives and not formally employed outside the home. Burkina Faso had the highest proportion of mothers who never attended school and who were farm workers or engaged in small businesses. Mothers in Burkina Faso consumed a diet low in diversity as compared with maternal diets in Bangladesh and Vietnam. Male heads of households engaged in a variety of occupations including farm work in all countries. Over 80% of households did not experience food insecurity in Bangladesh and Vietnam; data were not available on food security for Burkina Faso.

**Table 2:** Participant characteristics by country.

Characteristics	Bangladesh		Burkina Faso		Vietnam	
	Col%	*N*	Col%	*N*	Col%	*N*
Sample size (*n*)	100	998	100	1162	100	1002
**Child variables**						
Child's age group						
0–1 month	26.7	266	34.3	399	21.2	212
2–3 months	33.9	338	40.7	473	39.6	397
4–5 months	39.5	394	25.0	290	39.2	393
Child's sex						
Female	52.9	528	48.5	564	45.4	455
Male	47.1	470	51.5	598	54.6	547
Delivered at a facility, %	40.3	402	90.9	1056	99.4	996
**Maternal variables**						
Age group						
16–25 years	62.6	625	44.8	520	39.3	394
26–35 years	33.9	338	42.3	491	51.7	518
36–55 years	3.5	35	13.0	151	9.0	90
Education level: %						
Never attended school/in Class 1	12.6	126	74.4	865	0.4	4
Completed Class 1–5	30.8	307	17.6	205	8.5	85
Completed Class 6–9	43	429	0	0	39.0	391
Completed Class 10–12/higher	13.6	136	7.9	92	52.1	522
Occupation: %						
Housewife	81.5	813	0.0	0	12.9	129
Small business	15.8	158	24.4	283	15.7	157
Farm work	0	0	41.5	482	36.1	362
Other	2.7	27	34.2	397	35.3	354
Dietary diversity score, mean ± SD	7.6 ± 1.78		4.5 ± 1.85		8.2 ± 1.69	
**Paternal and household variables**						
Occupation: %						
Small business	31.2	311	27.3	317	22.7	227
Farm work	26.2	261	43.9	510	31.3	314
Other	42.7	426	28.8	335	46.0	461
Household food insecurity, %						
Food secure	83.0	823	na	na	80.7	799
Mild	4.9	49	na	na	8.7	86
Moderate	5.9	58	na	na	8.4	83
Severe	6.2	61	na	na	2.2	22

Abbreviations: Col%, column percentage; *N* and *n*, sample size; na, data not available; SD, standard deviation.

### 3.2 BF practices, knowledge and perceived social norms

Table 3 shows country differences in BF practices by intervention group and factors known to be drivers of BF practices including perceived social norms, self‐efficacy and knowledge of mothers. EBF and EIBF prevalences were higher in intervention areas compared with nonintervention areas across all countries. Among countries, EBF was higher in Bangladesh and Burkina Faso as compared with Vietnam, and EIBF was lowest in Burkina Faso. Colostrum feeding was high in both treatment groups in Bangladesh and Vietnam and lower in Burkina Faso. Pre‐lacteal feeding—a deleterious practice—was low in Bangladesh and Burkina Faso as compared with Vietnam. Perception of supportive social norms by mothers regarding the recommended practices was high. Self‐efficacy was higher in intervention areas regarding mothers' ability to follow recommended practices immediately after delivery and for 6 months of EBF. The total maternal knowledge scores for BF were the highest in Vietnam. Knowledge of the importance of EBF for children was almost universal in all countries in both intervention and nonintervention areas, but health benefits for mothers were less well‐known, except in Burkina Faso. Knowledge of colostrum was high in Bangladesh and Vietnam but varied across countries for EIBF and on‐demand feeding. Knowledge was higher in intervention areas as compared with nonintervention areas.

**Table 3:** Breastfeeding practices and associated factors by country.

Variables	Bangladesh				Burkina Faso				Vietnam			
	Nonintervention		Intervention		Nonintervention		Intervention		Nonintervention		Intervention	
	Col %	No.	Col %	No.	Col %	No.	Col %	No.	Col %	No.	Col %	No.
*BF Practices*												
**EBF** < **6 mo.**	***				***				***			
Yes	53	263	88	439	50.8	303	93.5	528	30.4	152	64.1	322
**EIBF**	***				***				***			
Yes	76.7	381	94.2	472	23.5	140	42.8	242	44.6	223	56.4	283
**Colostrum**					***							
Yes	96.4	479	97.2	487	77.3	450	95.2	537	96.8	484	98.2	493
**Pre‐lacteal feeding**	*				***							
Yes	5	25	1.6	8	10.4	62	1.6	9	99	495	99.6	500
*Associated factors*												
**Perceived social norms**	***				***				***			
‐Yes	72.4	360	86.3	432	86.1	514	94	531	99.6	498	100	502
**Self‐efficacy**	***								***			
BF after delivery	93.6	465	98.2	491	na	na	na	na	85.8	429	94.8	476
EBF for 6 mo.	73.8	367	91.2	456	na	na	na	na	34.2	171	67.1	337
Other^a^	80.7	401	92.6	463	na	na	na	na	29.0	145	62.2	312
**Knowledge** EBF												
‐Importance of EBF (child)	*											
Yes	96	477	98.4	493	100	597	100	565	97.8	489	98.4	494
‐Importance for mother									***			
Yes	20.7	103	21.6	108	98.3	587	98.2	555	63	315	74.1	372
‐Feed on demand					*							
Yes	97.8	486	97.4	488	57.8	345	48.8	276	78.6	393	82.3	413
‐Knowledge EIBF					***				*			
Yes	93.8	466	96	481	51.9	310	81.2	459	79.2	396	86.5	434
‐Colostrum					***				**			
Yes	89.1	443	92.2	462	51.9	310	71.9	406	93	465	97.6	490
Mother's total score, mean ± SD	4.07 ± 0.67		4.08 ± 0.56		4.17 ± 1.07		4.81 ± 0.86		4.65 ± 1.05		5.22 ± 0.90	

*Note*: The sample sizes may not add up to the total for some variables due to missing information, responses such as ‘do not remember’, ‘do not know’, ‘other’.

Abbreviations: BF, breastfeeding; EBF, exclusive breastfeeding; EIBF, early initiation of breastfeeding; SD, standard deviation.

^a^ The term ‘other’ refers to convincing other caregivers to practice EBF for her infant in Vietnam, and sufficient milk supply for 6 months in Bangladesh.

* *p* < = 0.05

** *p* < = 0.001

*** *p* < = 0.0001; comparisons are between treatment groups at endline.

Table 4 shows wealth‐ and education‐related inequalities in BF practices and associated factors. If the values are negative, the calculated indices suggest a bias favouring the disadvantaged (lower wealth or less education) and if the values are positive, the socioeconomically better‐off groups were favoured. We also note the magnitude of deviations from zero and identify whether they were greater or lesser in intervention and non‐intervention areas, and across the countries.

**Table 4:** Inequalities in breastfeeding practices, knowledge and perceptions by wealth and education.

Wealth Inequality						
Indicators	Bangladesh		Burkina Faso		Vietnam	
	Nonintervention	Intervention	Nonintervention	Intervention	Nonintervention	Intervention
*Breastfeeding practices*						
Exclusive BF	−0.037	−0.035	−0.028	−0.037	0.034	−0.001
Early initiation	−0.029	−0.038	0.055	0.086	−0.151	−0.162
Fed colostrum	0.022	−0.017	0.062	0.023	0.001	−0.012
Prelacteal feeding	0.000	0.021	−0.090	−0.071	0.000	−0.005
*Associated factors*						
Knowledge score	0.007	−0.003	0.011	0.000	0.025	0.011
Perceived social norms	0.093	0.033	0.040	−0.018	0.003	0.000

Education Inequality						
Indicators	Bangladesh		Burkina Faso^a^		Vietnam	
	Nonintervention	Intervention	Nonintervention	Intervention	Nonintervention	Intervention
*Breastfeeding practices*						
Exclusive BF	−0.027	0.027	na	na	0.046	0.092
Early initiation	−0.057	−0.020	na	na	−0.001	−0.067
Fed colostrum	0.015	−0.006	na	na	0.039	−0.004
Pre‐lacteal feeding	0.020	0.012	na	na	−0.006	−0.006
*Associated factors*						
Knowledge score	−0.005	−0.003	na	na	0.026	0.023
Perceived social norms	0.046	0.020	na	na	0.001	0.000

Abbreviations: BF, breastfeeding; EBF, exclusive breastfeeding; EIBF, early initiation of breastfeeding.

^a^ A continuous variable for education level was not available for Burkina Faso.

In all countries, the practice of EBF was pro‐poor in intervention areas; the pro‐poor EBF tendency in Burkina Faso and Vietnam was greater in intervention areas. EIBF was pro‐poor in Bangladesh and Vietnam and more pro‐poor in intervention compared with nonintervention areas. Colostrum feeding was pro‐poor in intervention areas in Bangladesh and Vietnam, and no clear inequality patterns were found for prelacteal feeding. Burkina Faso showed a pro‐rich bias in EIBF and colostrum feeding. Pre‐lacteal feeding—a deleterious practice—was more common among mothers from poorer quintiles in Burkina Faso. Knowledge scores favoured the better‐off in Vietnam, with almost neutral values for Bangladesh and Burkina Faso, and perceived social norms did not show clear patterns of inequalities.

Inequality in EBF by maternal education status (available only for Bangladesh and Vietnam) showed a bias towards more highly educated women. EIBF and colostrum feeding, however, favoured less‐educated mothers in intervention areas in Bangladesh and Vietnam. No clear patterns of education‐related inequalities were found for prelacteal feeding. Maternal knowledge scores favoured the more highly educated women in Vietnam and were close to neutral in Bangladesh. Perceived social norms did not show a clear pattern of inequality by maternal education status.

### 3.3 BF programme coverage

Table 5 provides the coverage of BF interventions and shows that a high proportion of women recalled counselling messages on EBF—69% in Bangladesh and 80% in Vietnam. Information on BF message recall was not available for Burkina Faso. The proportion who recalled counselling messages on EBF, EIBF, colostrum and how to position the baby for optimum BF was higher in intervention areas as compared with nonintervention areas. The number of IPC contacts was significantly higher in intervention areas as compared with nonintervention areas in all three countries. The proportions of mothers who received four or more BF counselling contacts were 7% in nonintervention areas as compared to 63.9% in intervention areas in Bangladesh, 23.7% in nonintervention areas as compared to 44.1% in intervention areas in Burkina Faso and 7.2% in nonintervention areas as compared to 24.1% in intervention areas in Vietnam. Public education/media coverage was higher in intervention as compared with nonintervention areas but less so than IPC coverage, possibly due to the inability of the programme implementers to limit exposure of public education/media in nonintervention areas. In Bangladesh, public education/media coverage was 46.9% and 52.5% in the two treatment areas, in Burkina Faso, 13.9% and 18.8% and in Vietnam, 50.4% and 58.2%, respectively. The combined exposure to both types of programme interventions was more than twofold to fourfold higher in intervention areas as compared with non‐intervention areas.

**Table 5:** Breastfeeding programme coverage by country and intervention group.

	Bangladesh				Burkina Faso				Vietnam			
	Nonintervention		Intervention		Nonintervention		Intervention		Nonintervention		Intervention	
Coverage indicators	Col %	No.	Col %	No.	Col %	No.	Col %	No.	Col %	No.	Col %	No.
**Interpersonal communication (IPC)**												
Messages at IPC contacts												
‐EBF for 6 months	***								***			
Yes	10.1	50	69.1	346	na	na	na	na	37.1	13	79.8	213
‐EIBF, initiate BF within <1 h.	***											
Yes	3	15	22	110	na	na	na	na	14.3	5	20.6	55
‐Give only colostrum	***											
Yes	4.8	24	31.1	156	na	na	na	na	11.4	4	10.5	28
‐How to position the baby	***											
Yes	0.8	4	26.3	132	na	na	na	na	14.3	5	26.2	70
No. of IPC contacts in the last 6 months	***								***			
At least 1	12.9	64	79	396	na	na	na	na	5.2	26	50.2	252
1	7.8	39	30.1	151	na	na	na	na	2.4	12	12.9	65
2	3.6	18	31.3	157	na	na	na	na	2	10	19.5	98
3 or more	1.4	7	17.6	88	na	na	na	na	0.8	4	17.7	89
Frequency of IPC contacts by category	***				***				***			
None	89.3	433	34.4	104	0	0	0	0	67.8	339	38	191
From 1 to 3	3.70	18	1.7	5	76.3	106	55.9	219	25	125	38	190
From 4 to 9	5.80	28	23.5	71	23	32	35.7	140	7	35	22.3	112
From 10 to 31	1.20	6	40.4	122	0.7	1	8.4	33	0.2	1	1.8	9
**Public education/media**												
Recall of messages by mothers					*				*			
Yes	46.9	233	52.5	263	13.9	83	18.8	106	50.4	252	58.2	292
**Total combined exposures**												
No. of platforms (IPC + public education/media)	***				***				***			
None, %	48.3	240	30.7	154	68.7	410	28.1	159	34.8	174	18.3	92
Any 1, %	46.1	229	46.5	233	25.5	152	55.6	314	47.8	239	43.2	217
Both, %	5.6	28	22.8	114	5.9	35	16.3	92	17.4	87	38.4	193
≥1, %	51.7	257	69.3	347	31.4	187	71.9	406	65.2	326	81.6	410

Abbreviations: EBF, exclusive breastfeeding; EIBF, early initiation of breastfeeding; IPC, interpersonal counselling; na, data not available.

* *p* < =0.05

*** *p* < =0.0001; comparison between treatment groups at endline.

Results of the inequality analyses for coverage indicators are shown in Table 6. IPC contacts for BF counselling slightly favoured the mothers in the poorest quintiles or were neutral (no bias) in all countries in intervention and nonintervention areas, but the magnitude of pro‐poor bias was lower in intervention areas particularly in Bangladesh. A reverse pattern was found in coverage of public education/media channels that reached more better‐off mothers in the three countries and in all treatment groups. However, the magnitude of the pro‐rich bias was slightly less in intervention as compared with nonintervention areas in Bangladesh, suggesting that efforts may have been made to engage public education/media platforms that reached disadvantaged mothers. In Burkina Faso and Vietnam, the pro‐rich bias was higher in intervention areas. The combined score for exposure to both IPC and public education/media channels for BF was slightly pro‐rich in intervention as well as nonintervention areas in all countries. When equality by maternal education was assessed, only one group—number of IPC contacts in intervention areas in Bangladesh—favoured the less educated. Both individually and when combined with IPC, public education/media favoured the more educated mothers slightly. However, the pro‐educated bias was less pronounced in intervention areas. Data on maternal education from Burkina Faso for this analysis were unavailable.

**Table 6:** Inequalities in breastfeeding programme coverage by wealth and education.

Wealth Inequality						
	Bangladesh		Burkina Faso		Vietnam	
Coverage indicators	Nonintervention	Intervention	Nonintervention	Intervention	Nonintervention	Intervention
IPC contacts	−0.208	−0.036	−0.011	−0.009	−0.017	0.001
Public education/media	0.390	0.189	0.111	0.151	0.146	0.199
Total no. of platforms	0.145	0.042	0.104	0.040	0.032	0.035

**Education inequality**						
	**Bangladesh**		**Burkina Faso** a		**Vietnam**	
**Coverage indicators**	**Nonintervention**	**Intervention**	**Nonintervention**	**Intervention**	**Nonintervention**	**Intervention**
IPC Contacts	0.141	−0.012	na	na	0.108	0.107
Public education/media	0.102	0.022	na	na	0.245	0.165
Total no. of platforms	0.037	0.006	na	na	0.091	0.066

Abbreviations: IPC, interpersonal counselling; na, not available.

^a^ a continuous variable for education level was not available for calculating EI in Burkina Faso.

## 4 DISCUSSION

Evidence on the beneficial health and development impacts of protecting BF practices in LMICs and HICs has grown (Victora et al., 2021). The establishment of global and national targets combined with rising threats posed by the pandemic, climate change and ill‐advised marketing of substitutes provide added urgency to expanding BF prevalence and supporting social norms, maternal knowledge, motivation and adoption particularly among disadvantaged groups (Pérez‐Escamilla et al., 2023; WHO, 2014). To our knowledge, this is the first time that three diverse LMIC programmes have been analysed using a common measure of inequality for BF practices and intervention coverage following exposure to interventions. The programmes had previously documented significant improvements in BF based on rigorous external evaluations (Cresswell et al., 2019; Menon et al., 2016). We needed to understand how the overall improvements had changed inequality in BF. There was concern that our BF programmes may reach the better‐off and more educated mothers, who may also face fewer barriers in adopting the recommended practices as suggested in previous papers (Lorenc et al., 2013; Santos et al., 2019; White et al., 2009).

Our results on wealth inequality in BF practices show that EBF in the three countries and EIBF in two out of three countries were pro‐poor in intervention areas at endline, indicating an advantage for less well‐off mothers. Programme design factors in the three study countries that may have protected BF practices among the less well‐off mothers include strengthening community‐based BF services in Bangladesh delivered through home visits by incentivized volunteers and providers, intensified community mobilisation in Burkina Faso to address social barriers and an explicit campaign to drive more mothers to primary health centres in Vietnam for BF counselling services. Similar interventions are also recommended to reduce inequity in health services, for example, outreach methods, placement of human resources in local areas or provided at the community level nearest to residents and financial or knowledge support that was found to be effective in reducing a range of disparities (Yuan et al., 2014). The Countdown to 2015 group has also provided evidence on interventions delivered at the community level that were more equitable across socioeconomic groups than those requiring access to fixed health facilities (Barros et al., 2012; Boerma et al., 2008).

Of concern in our results is a bias in favour of better‐off mothers in Burkina Faso in three critical BF practices, namely, EIBF, colostrum feeding and giving pre‐lacteal foods that are not recommended. We do not have qualitative data to unpack the factors that may have led to this divergence in the direction of inequality in Burkina Faso, with EBF favouring the poor, but EIBF, colostrum and no pre‐lacteal feeding favouring the better‐off mothers. One explanation is that care‐seeking for institutional deliveries has risen in Burkina Faso following removal of user fees that would have supported postpartum BF practices among disadvantaged mothers, and fixed facilities may not have adopted supportive BF interventions (Ilboudo & Siri, 2023). Alternatively, traditional practices surrounding the newborn may be more strongly held in Burkinabe communities than feeding practices such as EBF in the first 6 months. EBF to 6 months was pro‐poor in Burkina Faso and this could have been helped by the focus of the study programme as well as previous programmes that focused on EBF in the first 6 months intensively through community interventions (Cresswell et al., 2019).

When we analysed inequalities in BF practices by the educational status of mothers, more educated mothers had an advantage over less educated mothers in practicing EBF in intervention areas in Bangladesh and Vietnam, where we had data. This is consistent with trends in LMICs showing the protective effect of maternal education in BF. Women with no formal education have shown worsening BF indicators compared to women with primary and secondary or higher education (Neves et al., 2021). In Europe, Nepal and Indonesia, more mothers with high education were found to initiate BF as compared to those with low education and in some countries, continued EBF for longer (Acharya & Khanal, 2015; Nurokhmah et al., 2023; Sarki et al., 2019; Simpson et al., 2022; Skafida, 2009). The ability of educated mothers to understand the urgency of recommended BF practices, gain confidence and agency within families and communities to seek timely services and to comprehend and apply credible BF messaging to their situations may provide a more favourable environment, leading to better BF practices.

Our results on wealth inequality in programme coverage showed that IPC contacts for BF counselling slightly favoured the poorer mothers or had almost no bias in the countries in both intervention and nonintervention areas. However, the magnitude of pro‐poor bias was lower in intervention areas, indicating that well‐off mothers may have been reached slightly more successfully than poorer mothers. This is a concern and suggests the need for intentionally reducing social, economic or logistical barriers for less well‐off mothers to obtain BF counselling. Based on the programme context, this could be achieved by modifying existing services and/or adding new pro‐poor strategies.

The programmes in this study combined population‐wide interventions with interpersonal interventions, but reductions in inequalities in the three country programmes did not substantially contribute to a pro‐poor advantage. This is a serious gap as the deleterious health and development consequences of poor infant feeding are greater among the disadvantaged families and communities and less well‐off mothers should receive priority (Vilar‐Compte et al., 2022). Earlier reviews of interventions to reduce inequalities have shown the need for structural actions in addition to the various strategies used in our study programmes and may be a plausible reason behind our results (Gepkens, 1996). Structural actions for enabling BF include supportive workplace policies for mothers, removal of misleading marketing and promotion of breastmilk substitutes and reductions in unnecessary caesarean childbirth (Hobbs et al., 2016; Rollins et al., 2016). Some case studies in the literature provide examples of legislative measures in the Philippines and Pakistan, engagement of civil society in Mexico, behaviour change communication in Burkina Faso and humanitarian initiatives in Honduras and Pakistan (Hernández‐Cordero et al., 2022; Tomori, 2023). While our study countries have been participating in advocacy towards similar changes, greater efforts are needed in improving maternity care practices, code implementation and women's work conditions (Joyce et al., 2013; Nguyen et al., 2021; Nguyen, Cashin, Tran, Hoang, et al., 2022; Nguyen, Cashin, Tran, Vu, et al., 2022).

The limitations of our study include the use of self‐reported recall information from mothers to estimate the prevalence of BF practices and programme coverage. The study results only show inequality measures for the endline using cross‐sectional data and as such, there is no causality implied. Some indicators were different across countries and needed to be constructed with the information available, while some types of information were missing for a country, as shown in Table 1. The programme durations varied from 2 years in Burkina Faso to 4 years in Bangladesh and Vietnam.

In conclusion, the results indicate the need for introducing dedicated and highly focused actions for reducing inequalities in BF policies and programmes. This is a priority unfinished agenda for nutrition programming.

## AUTHOR CONTRIBUTIONS

Tina G. Sanghvi and Deepali Godha designed the study. Deepali Godha conducted the analysis. Tina G. Sanghvi and Edward A. Frongillo wrote the paper. All authors read and approved the final paper.

## CONFLICT OF INTEREST STATEMENT

The authors declare no conflict of interest.

## Data Availability

The data are publicly available through A&T's repository on Harvard Dataverse, and from the Alive & Thrive website (IFPRI & LSHTM, 2023). IFPRI, & LSHTM. (2023). Data Sets. Breastfeeding programme evaluations. Retrieved from: https://urldefense.com/v3/__https://www.aliveandthrive.org/en/our-data__;!!ELf_LxN3sEQ!eDHYHwPSAWNz0OHi5oEQDmhFlZUIaXR_mBwub9QYuXHTjSxUCUG577l4uKfefXCGYruyiqyZlyEGyqgEsK3P6UgRXhnjzWM$.
